# Predictive Analytics in Cardiothoracic Care: Enhancing Outcomes with the Healthcare Enabled by Artificial Intelligence in Real Time (HEART) Project

**DOI:** 10.46804/2641-2225.1195

**Published:** 2024-09-30

**Authors:** Felistas Mazhude, Robert S. Kramer, Anne Hicks, Qingchu Jin, Melanie Tory, Jaime B. Rabb, Mahsan Nourani, Douglas B. Sawyer, Raimond L. Winslow

**Affiliations:** a Department of Cardiovascular Services, MaineHealth Maine Medical Center, Portland, Maine; b MaineHealth Institute for Research, Portland, Maine; c Department of Anesthesiology and Perioperative Medicine, MaineHealth Maine Medical Center, Portland, Maine; d College Arts, Media and Design, The Roux Institute at Northeastern University, Portland, Maine; e College of Engineering, The Roux Institute at Northeastern University, Portland, Maine; f Khoury College of Computer Sciences, The Roux Institute at Northeastern University, Portland, Maine; g Bouve College of Health Sciences, The Roux Institute at Northeastern University, Portland, Maine; h Life Sciences and Medical Research Team, The Roux Institute at Northeastern University, Portland, Maine

**Keywords:** Cardiac surgical procedures, Postoperative complications, Forecasting machine learning, Clinical decision support systems

## Abstract

**Problem:**

Postoperative complications after cardiac surgery significantly impact both the short-term and long-term survival of patients. Cardiovascular diseases are a major health concern, accounting for 12% of health expenditures in the United States. A substantial number of patients with cardiovascular disease undergo invasive procedures, including cardiac surgery, and the incidence of postoperative complications is notable. This information underscores the need to effectively prevent postoperative adverse events to improve outcomes, reduce morbidity, shorten hospital stays, and lower health care costs.

**Approach:**

The Healthcare Enabled by Artificial Intelligence in Real Time (HEART) project is a collaborative effort involving clinicians from MaineHealth, industry experts from Nihon Kohden, and data scientists from the Roux Institute. The project aims to develop a real-time predictive analytics model as a decision support tool for clinicians in the cardiothoracic intensive care unit who care for patients after cardiac surgery. The team is using a supervised, closed-loop, machine learning design to train the model. The initiative involves collecting static and dynamic preoperative, intraoperative, and postoperative variables from a cohort of patients undergoing cardiac surgery at Maine Medical Center. These variables, including data on blood product transfusions and inotropic and vasoactive medications administered, are being transmitted from the electronic health record to a data warehouse. The model will predict the following adverse outcomes: acute kidney injury, renal failure, new onset postoperative atrial fibrillation, prolonged ventilation, reoperation, operative mortality, delirium, stroke, deep sternal wound infection, and extended hospital length of stay.

**Outcomes:**

The HEART team successfully established a data-collecting infrastructure. Data collection and validation are ongoing, with an emphasis on accuracy and completeness.

**Next Steps:**

The project will advance by developing a user-friendly, real-time interface, incorporating feedback from clinicians in the operating room and cardiothoracic intensive care unit to ensure practicality and acceptance of the technology. This interface will provide adverse outcome predictions in real time, support clinical decision-making, and become a regular part of patient care.

## 1. Problem

The impact of post-cardiac-surgery complications is well-documented, significant, and complex. The short-term and long-term survival of patients undergoing cardiac surgery in cardiothoracic intensive care units (CTICU) is negatively impacted by postoperative complications.^1,2^ Efforts must prioritize preventing adverse outcomes,^2^ thus improving patient outcomes.

According to the American Heart Association, coronary artery disease was the leading cause of death (40.3%) in 2021. Cardiovascular disease accounted for 12% of the United States’ health expenditure, more than any other major diagnostic group.^3^ Approximately 79% of patients with coronary artery disease undergo invasive procedures, which include stents and bypass surgery.^4^ These statistics suggest that a significant portion of patients with coronary artery disease choose surgical interventions, especially when symptoms are severe or their quality of life declines. Isolated coronary artery bypass graft accounts for more than half of adult cardiac surgeries done at most hospitals,^5^ and the rest are a combination of coronary artery bypass graft and valve, aortic, or isolated valve surgeries.

Patients undergoing cardiac surgery may experience the following postoperative complications (adverse outcomes) with notable incidences: acute kidney injury (16%), renal failure (2%), new onset postoperative atrial fibrillation (32%), prolonged ventilation (10%), reoperation (6%), operative mortality (3%), stroke (0.8% to 5.2% depending on the procedure type), delirium (26% to 52%), deep sternal wound infection (<1.0%), and short and prolonged hospital lengths of stay greater than 14 days (8%).^6–9^ Adverse outcomes after cardiac surgery can significantly increase morbidity,^10^ prolong hospital stays, and raise health care costs.^11,12^ For example, prolonged mechanical ventilation is a complication associated with a higher risk of respiratory complications and overall mortality.^13^ Also, postoperative delirium is associated with a greater risk of death, a higher likelihood of hospital readmission, and longterm declines in cognitive function and health-related quality of life.^14^ This project aims to develop a model that predicts such adverse outcomes early, potentially enabling timely interventions to prevent them.

### 1.1. Current risk prediction tools

Existing tools for predicting postoperative risk, such as the European System for Cardiac Operative Risk Evaluation (EuroSCORE) and the ACSD Operative Risk Calculator of the Society of Thoracic Surgeons (STS), are being widely used by cardiac surgeons.^15^ These tools use only preoperative static variables to predict complications, such as mortality, stroke, renal failure, prolonged ventilation, reoperation, and deep sternal wound infection.^15^ However, the intricacies of cardiac surgery and changes to patient status can alter the previously predicted outcomes with factors not incorporated in the STS and EuroScore models. The Healthcare Enabled by Artificial Intelligence in Real Time (HEART) project aims to address this gap by developing a dynamic predictive analytics model that considers real-time data from the electronic health record. These data include preoperative, intraoperative, and postoperative physiological variables. The team hypothesizes that this approach will significantly improve the predictive accuracy of risk models thus allowing health care teams to anticipate and prevent potential adverse outcomes for patients in the CTICU.

### 1.2. Proof of concept

Once trained with static and dynamic data, the predictive analytic machine learning (ML) algorithms recognize known and previously unknown patterns to determine the likelihood of adverse events. For instance, in an ICU setting, Liu et al.^16^ showed that a data-driven model could predict an impending septic shock before its clinical manifestation. This model identified a critical window, termed “early warning time,” during which clinicians could intervene before the patient’s condition deteriorated. Similarly, the HEART project aims to apply this concept by using ML techniques to predict adverse outcomes for patients in the CTICU who underwent cardiac surgery.

## 2. Approach

### 2.1. Model development and validation strategy

Artificial intelligence in medicine is evolving and could be an asset to help with many aspects of patient treatment.^17^

In the rural state of Maine, a collaboration between MaineHealth, Roux Institute, and Nihon Kohden industry experts comprising clinicians, data scientists, and information technologists is prospectively collecting more than 200 static and dynamic intraoperative and postoperative variables per patient.^18,19^ The team selected these variables based on expert opinions and a comprehensive literature review.^20^ The team will gather these data from approximately 1200 diverse adult patients who undergo cardiac surgery each year at Maine Medical Center. Additionally, the team is incorporating retrospective data to enhance the training of the model. This data includes approximately 9200 patients from the Northern New England (NNE) Cardiothoracic Surgery Consortium and EMR data from the MaineHealth Maine Medical Center CTICU.

Leveraging the team’s previous experience with developing predictive models, the team is using a supervised, closed-loop ML design to train the model. Data validation, ML techniques, and appropriate statistical metrics will be used to evaluate predictive accuracy and model performance.^21^ The model’s performance will be internally validated^22^ through methods such as bootstrapping. The team will repeatedly sample the training data during this process to ensure reliability and reduce overfitting.

### 2.2. Comparison of ML studies

Studies by Castela Forte et al.^23^ and Abbasi A et al.^24^ evaluated ML models that incorporate preoperative, intraoperative, and postoperative data to predict adverse outcomes after cardiac surgery. Castela Forte et al. focused on predicting mortality, whereas Abbasi et al. developed models to predict venous thromboembolism, stroke, and hemorrhagic reoperation. Both studies concluded that intraoperative data did not significantly improve model prediction. Conversely, Mori et al.^25^ found that adding intraoperative variables to preoperative variables improved predictions of adverse outcomes. The HEART project aims to study this controversial argument about the predictive power of intraoperative data. To do this, the team will develop 2 prediction models. Model 1 will integrate preoperative and intraoperative variables, and Model 2 will use preoperative variables to predict postoperative outcomes. The team will quantify the additive predictive power of intraoperative variables by comparing the area under the curve between Model 2 and Model 1.

### 2.3. Distinctive features of the HEART project

The HEART project distinguishes itself from other ML models through several advances. The team is collecting various physiological waveforms and clinical parameters from the electronic health record,^18,19^ including data on blood product transfusions and inotropic and vasoactive medications administered. Castela Forte et al.^23^ did not use these variables, but Mathis et al.^18^ and Khalpey et al.^19^ support their inclusion. The team expects that this comprehensive data integration will enhance the predictive accuracy of the model.

The HEART project model targets to predict 11 adverse outcomes: acute kidney injury, renal failure, new onset postoperative atrial fibrillation, prolonged ventilation, reoperation, operative mortality, stroke, delirium, deep sternal wound infection, and short and prolonged hospital lengths of stay greater than 14 days. This broader adverse outcome focus sets the HEART project apart from other models that have a narrower scope.

### 2.4. Real-time predictive capabilities

The HEART project is distinguished by its real-time predictive capabilities. Existing models have not offered real-time feedback and used a limited number of variables for training. The HEART project will provide real-time adverse outcome predictions, support clinical decision-making, and become part of the standard of care when validated.

## 3. Outcomes

The HEART team has successfully set up a robust data-collection infrastructure. Patient data collection and validation focusing on accuracy and completeness are ongoing. This rigorous approach to data quality aims to enhance the reliability of the model’s predictions.

## 4. Next steps

**Fig. 1.**
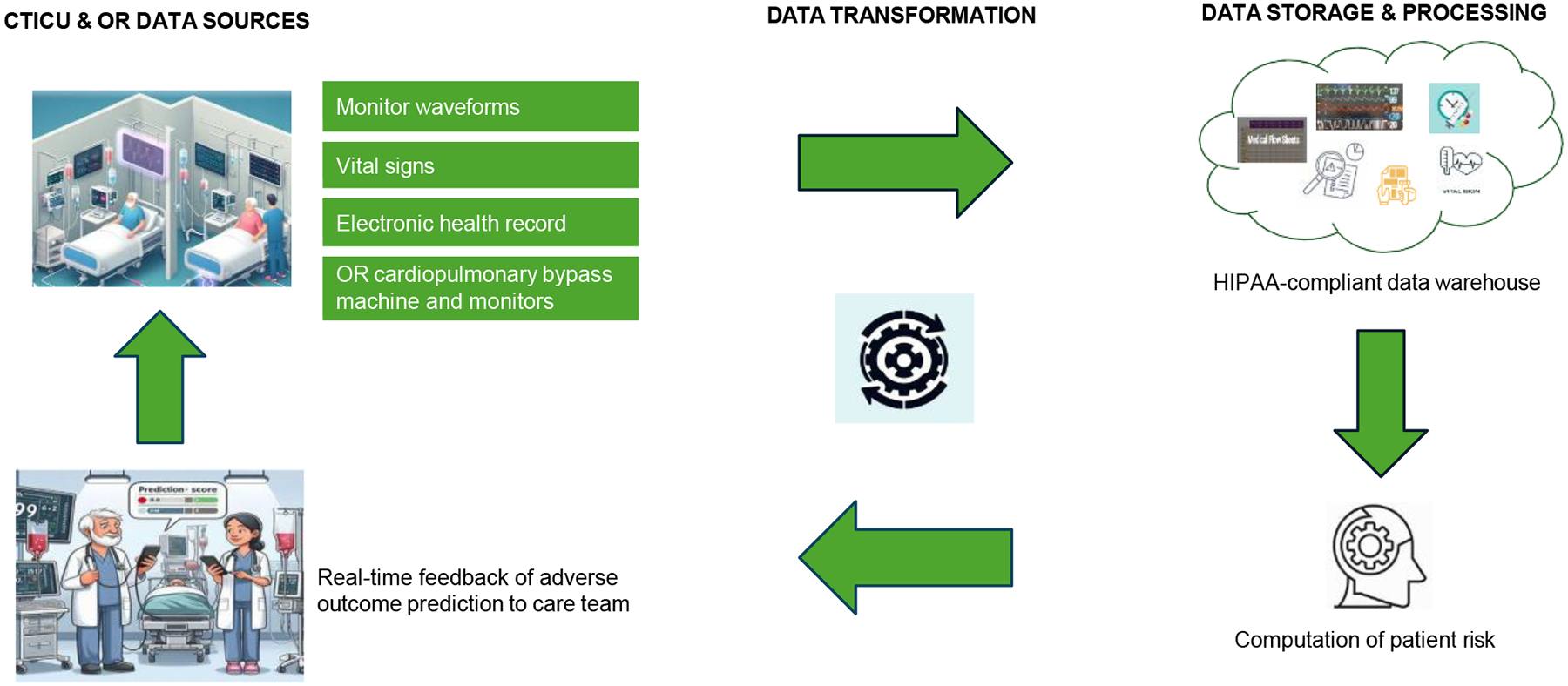
Streamlined data flow from the Cardiothoracic Intensive Care Unit (CTICU) and Operating Room (OR) sources to a Health Insurance Portability and Accountability Act (HIPAA)-compliant data warehouse for real-time patient adverse outcome computation and feedback of the probability of adverse outcomes to the care team.

Most of the steps in model development will occur concurrently. To develop a user-friendly real-time interface/platform, the team will gather input from clinicians in the OR and CTICU to ensure the technology’s practicality and acceptance within the clinical environment. The team will deploy the interface to provide clinicians with adverse outcome predictions based on ML and in real time, enhancing informed clinical decision-making (Fig. 1).

### 4.1. Practical application of predictive analytics in the clinical workflow

The HEART project model will serve as a decision support tool rather than a prescriptive one. The care team, guided by clinical judgment without the model’s direction on specific interventions, can initiate preemptive measures that may reduce the severity or incidence of adverse outcomes. For instance, when the model predicts that the patient has a higher risk for acute kidney injury, the care team can respond by adjusting nephrotoxic medications, initiating diuretics, or preparing for renal replacement therapy,^26^ where applicable.

In conclusion, the HEART project represents a promising advancement in cardiac surgery by introducing a dynamic, real-time predictive model that adapts to changing patient conditions. This approach has the potential to transform patient care in the CTICU by providing clinicians with timely adverse outcome predictions, enabling proactive interventions, and potentially improving overall patient outcomes.
